# Laboratory Diagnosis of Human Rabies: Recent Advances

**DOI:** 10.1155/2013/569712

**Published:** 2013-11-14

**Authors:** Reeta Subramaniam Mani, Shampur Narayan Madhusudana

**Affiliations:** Department of Neurovirology, WHO Collaborating Centre for Reference and Research on Rabies, National Institute of Mental Health and Neurosciences (NIMHANS), Bangalore 560029, India

## Abstract

Rabies, an acute progressive, fatal encephalomyelitis, transmitted most commonly through the bite of a rabid animal, is responsible for an estimated 61,000 human deaths worldwide. The true disease burden and public health impact due to rabies remain underestimated due to lack of sensitive laboratory diagnostic methods. Rapid diagnosis of rabies can help initiate prompt infection control and public health measures, obviate the need for unnecessary treatment/medical tests, and assist in timely administration of pre- or postexposure prophylactic vaccination to family members and medical staff. Antemortem diagnosis of human rabies provides an impetus for clinicians to attempt experimental therapeutic approaches in some patients, especially after the reported survival of a few cases of human rabies. Traditional methods for antemortem and postmortem rabies diagnosis have several limitations. Recent advances in technology have led to the improvement or development of several diagnostic assays which include methods for rabies viral antigen and antibody detection and assays for viral nucleic acid detection and identification of specific biomarkers. These assays which complement traditional methods have the potential to revolutionize rabies diagnosis in future.

## 1. Introduction

Rabies, one of the oldest and most feared zoonotic diseases known to mankind, is an acute, progressive, and almost fatal encephalomyelitis caused by the *Rabies virus *(RABV) and other *Lyssavirus* species of the family Rhabdoviridae. 

Despite the lack of accurate data on the global burden of neglected tropical diseases, the estimates of direct mortality due to rabies, transmitted most commonly through the bite of a rabid animal, are among the highest. The annual number of human rabies deaths globally, in 2010, is estimated to be 61,000 (95% CI 37,000–86,000), with the vast majority of deaths (84%) occurring in rural areas. The estimated annual cost of rabies is US$ 6 billion (95% CI, 4.6–7.3 billion), with almost US$ 2 billion due to lost productivity after premature deaths and a further US$ 1.6 billion spent directly on postexposure prophylaxis [1].

Most of the human deaths due to rabies occur in Asia and Africa. Estimates of human mortality due to endemic canine rabies in Asia and Africa annually exceed 30,000 and 23,000, respectively [2]. In Latin America and the Caribbean, a substantial success in canine rabies control and a reduction in human rabies transmitted by dogs has been achieved during the past two decades. However, the incidence of bat rabies has reportedly increased, probably resulting in more human cases and livestock losses [3]. 

Canine rabies has been eliminated from western Europe, Canada, the United States of America (USA), Japan, Malaysia, and a few Latin American countries. Australia is free from carnivore rabies, and many Pacific Island nations have always been free from rabies and related viruses. In these areas, human deaths from rabies are restricted to people exposed while living or travelling in areas endemic for canine rabies [1, 4]. However, the cost of rabies prevention in many countries where wildlife rabies or bat rabies viruses circulate is substantial. About one to eight human rabies deaths occur annually in the USA as a result of wildlife rabies and an estimated US$ 300 million are spent per annum for rabies prevention [1, 5].

Laboratory diagnosis and surveillance for animal and human rabies are severely constrained in much of the developing world where rabies is endemic. The true disease burden and public health impact due to rabies remain underestimated due to lack of simple, sensitive, and cost-effective laboratory methods for rabies diagnosis. This may be one of the important reasons why rabies remains a neglected zoonotic disease in many developing countries in Asia and Africa [6, 7]. 

## 2. Need for Laboratory Diagnosis in Human Rabies Cases

Two distinct forms of rabies—furious and paralytic—are recognized in humans. Diagnosis of the classical furious (encephalitic) form, which constitutes about 80% of human rabies cases, is based on its distinctive clinical signs and symptoms and rarely poses diagnostic difficulties. However laboratory assistance may be required in some cases wherein characteristic clinical features like aerophobia or hydrophobia are lacking. In clinical practice, the paralytic or atypical forms, which constitute about 20% of human rabies cases, pose a diagnostic dilemma. These cases are often clinically indistinguishable from Guillain-Barre syndrome (GBS) and also need to be differentiated from neuroparalytic complications due to Semple-type antirabies vaccine which is still being used in few countries like Mongolia, Myanmar, and Pakistan [8–11]. The situation is further compounded by lack of history of animal bite, psychiatric or other atypical clinical manifestations, unavailability of a definitive diagnostic test for GBS, and limited availability of tests for antemortem diagnosis of human rabies [9].

Rapid diagnosis of rabies is vital for initiating prompt and appropriate infection control and public health measures. Early diagnosis can obviate the need for unnecessary treatment and medical tests and also help in prognostication, institution of barrier nursing, timely administration of pre- or postexposure prophylactic vaccination to family members of the patient and the treating medical and nursing staff, and case closure and grief counselling with family members. Laboratory tests negative for rabies can indicate the presence of another infectious agent or a noninfectious aetiology, and assist in appropriate medical management. Laboratory diagnosis of rabies can also help specific characterization of the causative agent and suggest the potential source of infection, especially when a history of exposure to an animal is lacking, and identification of other individuals who may have been exposed to the same source of infection [1, 9, 12].

Reports of rabies being acquired through organ transplants [13–15] highlight the need for organ donor screening for rabies, especially in donors with acute progressive encephalitis of unexplained aetiology, and presence of other risk factors for rabies.

Although human rabies is known to be almost 100% fatal, the reported survival of a teenager who developed rabies following a bat bite in USA using the “Milwaukee Protocol” in 2005 [16] has revived interest in the medical community to attempt experimental therapeutic approaches. The potential for treatment provides an additional impetus to try to make the diagnosis as soon as possible [17] and hence antemortem laboratory diagnosis has assumed greater significance in recent years. Studies to identify management protocols, procedures for immunomodulation, and new medications, including antiviral drugs, are encouraged by the recent WHO expert consultation [1].

Continual surveillance and laboratory confirmation in clinically suspected cases of rabies are imperative in countries which have recorded a decline in human rabies cases in recent years (e.g., Sri Lanka, Thailand) and are working towards rabies elimination in the near future. Geographical boundaries cannot restrain the rabies virus; as long as foci of wildlife or canine rabies exist anywhere, and international travel and global trade of livestock, pets, and wildlife continue, the threat of reintroduction of rabies exists even in countries which have been rabies-free for many years. For instance, Bali, an Indonesian island, was considered rabies-free until late November 2008. However, an island-wide rabies outbreak has since occurred and rabies has been confirmed in both dogs and humans, causing 141 human deaths by the end of 2012 [1].

## 3. Conventional Diagnostic Tests for Rabies: Advantages and Limitations

Laboratory techniques in rabies were started as early as 1800 BC when for the first time Zinke demonstrated that the infection could be transmitted to a normal animal after inoculating with saliva from a rabid animal. The landmark discovery of Negri bodies by Adelchi Negri in 1903 and demonstration of their diagnostic significance by his wife Lina Negri-Luzzani in 1913 paved the way to laboratory confirmation of rabies. 

A definitive diagnosis of rabies can be made only with the appropriate laboratory methods. The basic techniques are described in the WHO publication *Laboratory Techniques in Rabies *[18] *and the OIE Manual of Diagnostic Tests and Vaccines for Terrestrial Animals *[19].

### 3.1. Direct Microscopy: Histological Identification of Characteristic Cell Lesions

Infected neuronal cells reveal aggregates of viral particles “Negri bodies” which are intracytoplasmic inclusion bodies specific to rabies encephalitis, demonstrated by histological tests (Seller's Technique) on smears taken from various areas of the brain. Negri bodies vary in size from as small as 3 *μ*m to as large as 30 *μ*m and are generally circular or oval and deeply eosinophilic with characteristic basophilic granules, often arranged in the form of a rosette, within the eosinophilic matrix. 

Though it is a simple, rapid test, Seller's method on unfixed tissue smears has a very low sensitivity and is only suitable for fresh specimens. Techniques that stain sections of paraffin embedded brain tissues are time consuming, less sensitive, and more expensive. Histological techniques are much less sensitive than immunological methods, especially in the case of autolysed specimens, and are no longer recommended for primary diagnosis, both in humans and animals [1, 18, 19].

### 3.2. Demonstration of Viral Antigen

#### 3.2.1. Fluorescent Antibody Technique (FAT)

**Figure 1:**
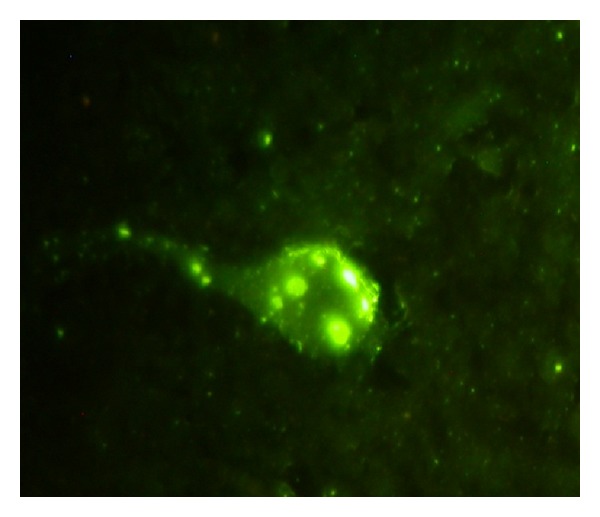
Fluorescent antibody technique (FAT) on human brain smear positive for rabies.

The most widely used test for postmortem rabies diagnosis is the fluorescent antibody test (FAT), which is recommended by both World Health Organization (WHO) and World Organization for Animal Health (OIE). Developed by Goldwasser and Kissling in 1957, this test is still the gold standard for rabies diagnosis [20, 21]. It involves demonstration of the rabies virus nucleoprotein antigen (N) in fresh brain smears of a suspected rabies case by using immunofluorescence technique (Figure 1). It can also be used to confirm the presence of rabies antigen in cell culture or in brain tissue of mice that have been inoculated for diagnosis. The specificity and sensitivity of the test almost approach 99% in an experienced laboratory and results are available within a few hours. 

Reliable results are obtained only when fresh brain tissue is used; however, FAT can also be applied to specimens preserved in 50% glycerol saline after rigorous washing of the specimens with normal saline. If the specimen has been preserved in a formalin solution, FAT may be used only after the specimen has been treated with a proteolytic enzyme [22]. However, the FAT on formalin-fixed and digested samples is always less reliable and more cumbersome than when performed on fresh tissue [23]. Partially decomposed brains are not suitable for this test as it is very difficult to differentiate specific fluorescence due to N antigen from nonspecific fluorescence which may result from bacterial contamination. 

Obtaining a postmortem brain biopsy/autopsy continues to be a challenge due to religious, cultural, and other factors, with declining rates of autopsy being a common problem in both developed as well as developing countries. Availability of methods of antemortem diagnosis of rabies in samples other than brain tissue is very critical and would circumvent the need for invasive brain biopsies/autopsy and the associated logistics and safety procedures. 

FAT can also be performed on corneal smears and nuchal skin biopsy in suspected cases; however it has been found to have limited reliability and low sensitivity for antemortem diagnosis of rabies [24, 25]. Besides, FAT on corneal impressions is not recommended as a routine test because of the risk of corneal scarification, particularly in patients with encephalitis and not rabies [1]. 

Furthermore, the need for an expensive fluorescence microscope, which requires maintenance as well as skilled personnel to interpret the test, limits its use in many developing countries.

#### 3.2.2. Rapid Rabies Enzyme Immunodiagnosis (RREID)

**Figure 2:**
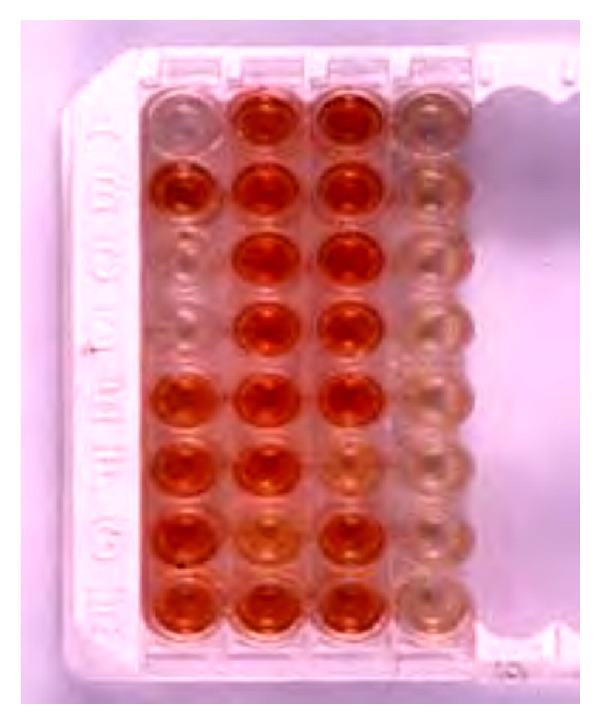
Diagnosis of rabies by the Rapid Rabies Enzyme Immunodiagnosis (RREID) technique. Note the dark brown colouration obtained with rabies positive brains in comparison to negative brains which appear colourless.

The rabies N antigen can also be detected by applying immunohistochemical techniques as well as enzyme immunoassays. An ELISA-based technique was developed by Perrin et al. in 1986 which is known as rapid rabies enzyme immunodiagnosis (RREID) [26]. This technique is based on capturing rabies N protein in a brain homogenate by a polyclonal or monoclonal anti-N antibody coated on the solid phase. Subsequently, the captured antigen is detected by adding peroxidase conjugated monoclonal or polyclonal antibody raised in a different species or even better by the addition of biotinylated N antibody followed by streptavidin peroxidase and colour development with o-phenylenediamine dihydrochloride (OPD) and hydrogen peroxide. In various studies, the test is found to be as sensitive and specific as FAT [27, 28] (Figure 2). An added advantage is that partial decomposition of the brain will not affect the test result. A limitation of the test is requirement of brain tissue, which precludes its use in antemortem diagnosis. 

### 3.3. Virus Isolation

Virus isolation is required for confirmatory diagnosis, especially when FAT gives an uncertain result and more importantly for molecular characterization of viruses in a geographical location and for tracing the origin of the virus if rabies occurs in a rabies-free area. Two techniques can be employed for this purpose: the mice inoculation technique (MIT) and rapid tissue culture infection test (RTCT) [29, 30]. 

#### 3.3.1. Mouse Inoculation Test

Three-to-ten mice, 3-4 weeks old (12–14 g), or a litter of 2-day-old newborn mice, are inoculated intracerebrally with the clarified supernatant of a 10–20% (w/v) homogenate of brain material in an isotonic buffered solution containing antibiotics. The inoculated mice are observed daily for 28 days; they develop typical signs and symptoms of rabies any time after 5–7 days depending on the incubation period. These consist of initial ruffling of hair, hunch back, and dragging hindlimbs followed by paralysis of hind- and forelimbs. Further confirmation of the diagnosis can be made by extracting the brain of the diseased mouse and subjecting this to FAT. 

The disadvantage of MIT is the long interval before a diagnosis can be made since the inoculated mice need to be kept under observation for 28 days as some wild viruses may have a very long incubation period. If cell culture facilities exist in the laboratory, consideration should be given to replacing the mouse inoculation test with cell culture whenever possible as it avoids the use of live animals, is less expensive, and gives more rapid results. However, advantages of MIT are that when the test is positive, a large amount of virus can be isolated from a single mouse brain for strain identification purposes and that it can be easily and practicably applied in situations where skills and facilities for other tests (e.g., cell culture) are not available [19]. 

#### 3.3.2. Rapid Tissue Culture Infection Test (RTCT)

**Figure 3:**
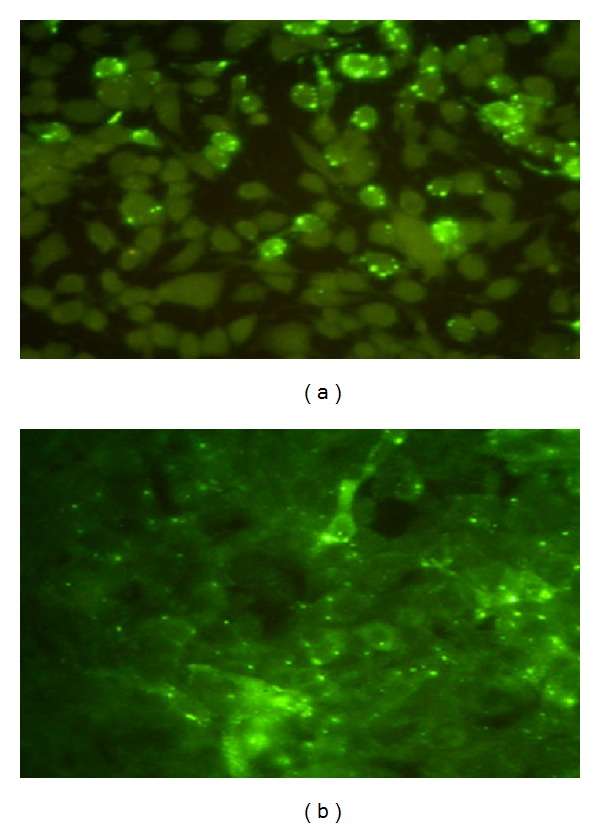
Virus isolation in cell culture: street virus infected Neuro-2a (a) and HEK 293 (b) stained with fluorescent antibody technique (FAT).

As compared to MIT, virus isolation in cell culture is fast and results can be given in 24–48 hours. The cell lines most suitable for virus isolation are of neural origin and the most commonly used cell line is the murine neuroblastoma cell line Neuro-2a. Other cell lines which are used but may not be as sensitive as Neuro-2a include chicken embryo-related (CER) and baby hamster kidney (BHK 21) cells. The suspect clinical specimen or the brain homogenate is inoculated onto the cells grown in a shell vial or 96-well plates, incubated for 24 h and stained by direct FAT after acetone fixation. Recently, a new cell line, human embryonic kidney cell line (HEK 293), was evaluated for isolation of both fixed and street viruses and was found to be as sensitive and specific as Neuro-2a cell line [31] (Figure 3). RTCT is a faster and cheaper alternative to MIT; however it can be performed only in laboratories with cell culture facilities as well as a fluorescent microscope.

### 3.4. Demonstration of Antibodies

The demonstration of antibody in the serum in the absence of a history of vaccination for rabies or in CSF offers indirect evidence of rabies infection. Interpretation of test results may be difficult since the host immune response may vary among individuals. The negative predictive value of serological tests for rabies diagnosis is considered poor [32]. Serological testing is rarely useful for antemortem diagnosis because of late seroconversion and the high mortality rate of host species but may assist in diagnosis of paralytic rabies, where the survival is relatively longer. Serological techniques are, however, very useful for assessing seroconversion following vaccination and for epidemiological studies. 

As neutralizing antibodies are considered a key component of the adaptive immune response against rabies virus, virus neutralization (VN) assays in cell cultures are prescribed tests for checking vaccination responses. Results are expressed in international units relative to an international standard antiserum. The Mouse Neutralization Test (MNT), Rapid Fluorescent Focus Inhibition Test (RFFIT), and the Fluorescence Antibody Virus Neutralization Test (FAVN) have been described for this purpose. The widely used virus neutralization test in mice (MNT) developed in 1935 by Webster and Dawson [33, 34] is no longer recommended by the WHO or OIE.

The adaptation of the “challenge virus standard (CVS) strain” of rabies virus to grow in cell culture has led to the development of alternative assays in which the replication of nonneutralized virus in culture is detected by the observation of plaques, infected cells stained with fluorescent antibody, or the colour developed with enzyme-labeled anti-rabies antibody. The RFFIT is one of the most widely used substitutes to the Mouse Neutralization Test (MNT) and is a rapid test that requires only 20 hours for completion and is slightly more sensitive than the MNT for detecting virus neutralizing antibodies (VNAs) in postvaccinal sera [35, 36]. The FAVN test, which is an adaptation of RFFIT, was developed in 1998 and results obtained showed good agreement with the MNT and RFFIT [37]. A modification of this test in which the monoclonal anti-rabies antibodies and a peroxidase anti-mouse conjugate were used instead of a fluorescein-conjugated anti-rabies antibody is also reported [38]. 

In a recent laboratory study on diagnosis of human rabies, rabies viral neutralizing antibodies were detected using RFFIT in 4/11 (36.3%) CSF samples received for antemortem diagnosis and 7/13 (53.8%) CSF samples obtained postmortem, which represents the terminal stage of illness. An inverse correlation was found between detection of neutralizing antibodies and presence of viral RNA in CSF samples [39]. Antibody testing is also a useful tool to monitor the immune response within the central nervous system and thus a possible clearance of the rabies virus in patients who are treated with the “Milwaukee Protocol” or other experimental interventions in future [40]. 

The RFFIT is considered the gold standard assay and has been used to estimate the titre of rabies virus neutralizing antibodies for several years. However the test requires trained manpower, cell culture and fluorescent microscopy facilities, and adequate biosafety measures to handle live virus. The RFFIT is sensitive to cytotoxicity in poor-quality sera [41] and nonspecific inhibitors of virus in sera may produce false positive results. 

## 4. Newer Diagnostic Tests for Rabies

### 4.1. Demonstration of Viral Antigen

#### 4.1.1. Direct Rapid Immunohistochemical Test (dRIT)

**Figure 4:**
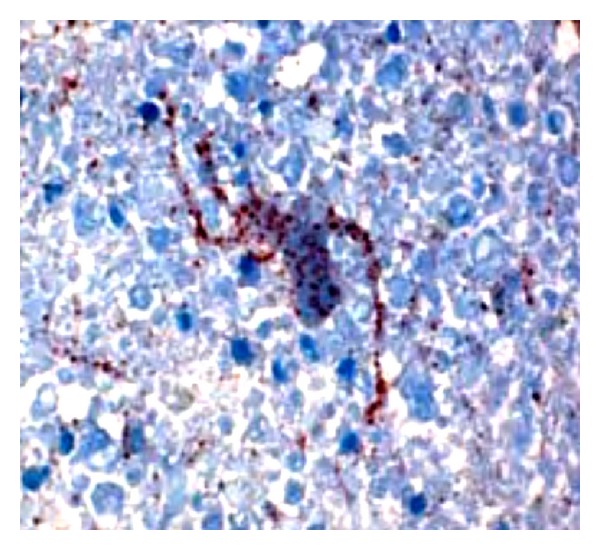
The Direct Rapid Immunohistochemical Test (dRIT) technique done on a human brain positive for rabies. Note the presence of brownish red particles in a neuron spreading along dendrites and axon.

One of the most significant developments in recent years is the development and evaluation of a rapid immunohistochemical test called Direct Rapid Immunohistochemical Test (dRIT) developed at Centers for Disease Control (CDC), Atlanta, USA [42]. The test is based on detecting rabies N protein in suspected brain smears fixed in buffered formalin using a cocktail of highly concentrated and purified biotinylated monoclonal antibody to N protein followed by addition of streptavidin peroxidase and substrate colouring reagent (H_2_O_2_ and amino ethyl carbazole). The rabies N antigens, if present, are found as brownish red clusters within the neuron, along the axons and scattered all over the brain smears (Figure 4). The whole test procedure takes less than one hour and has the advantage of applicability under field conditions as expensive fluorescence microscope is not required. The test has been evaluated under field conditions in Tanzania and was found to be 100% sensitive and specific compared to FAT. The test could be successfully performed on samples preserved in glycerol solution for 15 months or frozen for 24 months and in variable conditions of preservation. This increases its suitability for use in field conditions in developing countries, where cold storage facilities may not be available [7]. The test has also undergone extensive evaluation in other countries [43–45] and 100% correlation was found with FAT. 

Though refrigerated reagent storage is required for dRIT, one of the main advantages of this newly developed test is the relative ease of interpretation using an ordinary light microscope. This simple test will enable developing countries to enhance rabies epidemiologic surveillance at greatly reduced cost and without the need for prohibitively expensive fluorescent microscopic equipment along with the expertise and financial input needed to maintain them. The cost-effectiveness of the dRIT will allow knowledge and technology transfer to remote areas of the developing world where rabies incidence data are difficult to obtain. It could be also valuable in guiding decisions regarding rational use of postexposure prophylaxis for rabies [7, 44].

The WHO recommends further development of direct rapid immunohistochemistry tests as an alternative to the direct fluorescent antibody test for improved decentralized laboratory-based surveillance [1]. However, one major factor that needs consideration before advocating laboratories in developing countries to adopt the dRIT for rabies diagnosis is the uninterrupted supply of the critical reagents (anti-N monoclonal antibodies) which are presently available only through CDC, Atlanta. Commercialization of the reagents or feasibility of indigenous production in selected laboratories in developing countries should be considered [43].

#### 4.1.2. Indirect Rapid Immunohistochemistry Test (IRIT)

Recently, an indirect rapid immunohistochemistry test (IRIT), for the detection and differentiation of rabies virus (RABV) variants, was evaluated by traditional light microscopy. Fresh frozen brain touch impressions or cell culture monolayers fixed in buffered formalin are stained with a panel of murine anti-nucleoprotein monoclonal antibodies (mAb-N) and commercially available biotin-labeled goat anti-mouse antibody. Ninety-six (96) RABV isolates, representing 20 RABV variants previously determined by antigenic typing using a panel of mAb-N and the indirect fluorescent antibody test (IFA), and genetic sequence analysis were characterized by IRIT. IRIT results revealed distinct reactivity patterns associated with current and historical RABV reservoir hosts similar to IFA test and genetic sequence analysis.

IRIT does not require specialized equipment and can be performed in a field setting. Further, commercially available labeled secondary antibodies permit the use of a standard panel of unlabeled primary mAbs, without the need for fluorescence microscopy. IRIT can therefore be used as a cost-effective diagnostic test, which can also help to study the prevalence, distribution, and transmission of rabies virus among reservoir hosts in rabies enzootic areas [46].

#### 4.1.3. Immunochromatographic Techniques

Another recently described method for the detection of rabies virus antigen from postmortem samples is the rapid immunodiagnostic test (RIDT), a useful method for rabies diagnosis without the need for laboratory equipments. This immunochromatographic lateral flow strip test is a one-step test that facilitates low-cost, rapid identification of viral antigen. Briefly, processed suspect material is added to the test device and conjugated detector antibodies attached to two different zones on a membrane indicate the presence of viral antigen [47]. Another test for rabies viral antigen detection, using the same principle, was developed using a combination of purified polyclonal and monoclonal antibodies and evaluated with dog saliva samples [48]. A simple and rapid immunochromatographic test kit for rabies diagnosis developed using monoclonal antibodies which recognize epitope II and III of the nucleoprotein of rabies virus has also been evaluated as a rapid screening test for rabies [49]. An improved version of the same has also been recently developed for animal and human rabies diagnosis and found to be a reliable, user friendly, rapid, and robust test to be used in laboratories with modest infrastructure. Though this test showed high sensitivity, it had a low specificity for human brain samples and hence unsuitable for human rabies diagnosis [50]. Though tests based on immunochromatographic techniques can be used as a rapid screening test in animals, they need to undergo considerable improvement and evaluation on human clinical samples, before they can be recommended for use in the diagnosis of human rabies.

#### 4.1.4. Other Antigen Detection Assays

A simple, reliable and rapid sandwich ELISA (WELYSSA) can be used for detection of *Lyssaviruses* belonging to all seven genotypes circulating in Europe, Africa, Asia, and Oceania [51, 52]. Other tests for rabies viral antigen detection include a dot-blot immunoassay for brain tissues [53] and an enzyme immunoassay for rapid diagnosis of rabies in humans and animals [54]. 

The colorimetric enzymes used in rabies viral antigen detection are usually coupled to an antibody by chemical means using cross-linking reagents, which can lead to heterogeneous conjugates, sometimes with reduced activity and specificity. Chimeric bifunctional molecules in which the variable domains of an antibody are linked to unrelated protein tracers by genetic engineering can help circumvent this problem. A recent study described the successful production of a bifunctional chimeric protein based on alkaline phosphatase-fused anti-rabies virus glycoprotein scFv antibody fragment. It was found to be a novel in vitro tool for detecting rabies viral antigen in brain smears [55].

### 4.2. Nucleic Acid Detection Techniques

Nucleic acid amplification and detection techniques have revolutionized the diagnosis of rabies in recent years and have an important role in the antemortem diagnosis of human rabies. Currently, several polymerase chain reaction (PCR) based assays have been evaluated as an adjunct to conventional tests for antemortem and postmortem rabies diagnosis. Most assays target the highly conserved rabies viral nucleoprotein gene for amplification. 

#### 4.2.1. Reverse Transcriptase PCR (RT-PCR)

Several conventional gel-based reverse transcriptase PCR (RT-PCR) assays with nested/heminested protocols for detection of rabies viral RNA on clinical samples have been described [12, 56–60]. The amplicons generated in these assays can be sequenced for further virus characterization and phylogenetic analysis. However a major drawback of these assays is the risk of cross-contamination, which precludes their routine use for diagnosis of human and animal rabies.

#### 4.2.2. Real-Time PCR

Real-time PCR based assays allow for the detection and quantification of genome copies and a considerable reduction in cross-contamination is achieved due to the “closed-tube” nature of these assays. Real-time PCR using the SYBR Green chemistry has been evaluated on human saliva samples for antemortem rabies diagnosis [61] and as a universal real-time assay for the detection of *Lyssaviruses* [62]. Though these assays are promising, extreme care is needed to ensure specificity [63]. Real-time PCR assays using the TaqMan fluorogenic probes, however, ensure a high specificity because of the intrinsic hybridization reaction [39, 63–66], have a wide range of detection, and are 10–1000 times more sensitive than traditional nested RT-PCR [63, 64]. In a recent study, real-time TaqMan PCR for viral RNA was positive in 5/11 (45.4%) CSF samples, 6/10 (60%) nuchal skin biopsies, and 6/7 (85.7%) saliva samples, obtained antemortemly from patients with clinically suspected rabies. At least one clinical sample (CSF/skin/saliva) was positive by real-time TaqMan PCR in 11/13 (84.6%) patients; combined with rabies viral neutralizing antibody detection in CSF, antemortem rabies diagnosis could be achieved in all 13 (100%) cases [39]. 

Despite several advantages, viral genetic heterogeneity may prove to be an impediment to the development of TaqMan probe based PCR since mismatches between the target and the probe can lead to false negative results or decreased sensitivity [64, 65]. However mismatches on primer and/or probe binding sites did not affect the amplification or detection in several other studies [66].

*Other Molecular Assays*. The nucleic acid sequence-based amplification (NASBA) technique uses three enzymes to generate multiple copies of the RNA under isothermal conditions. The automated NASBA enables easy and rapid testing of samples and has been reported to have a higher sensitivity than conventional PCR assays for detection of rabies viral RNA in antemortem saliva and CSF samples [67]. Loop-Mediated Isothermal Amplification (LAMP) is an alternative method of amplification of DNA with high specificity and efficiency and without the need for thermal cycling, which has been used for rabies diagnosis [68, 69]. Since this method does not have the technological requirements of thermal cycling used in RT-PCR, it can be used to develop surveillance protocols where testing can take place in the field or in less sophisticated laboratories [32].

Nucleic acid detection tests can be performed on a range of biological samples like CSF, saliva, tears, urine, skin biopsy, extracted hair follicles, and brain tissue for antemortem and postmortem diagnosis of human rabies. These assays have been found beneficial for diagnosis of rabies in decomposed and archival samples [39, 70–72] and have an important role in retrospective diagnosis and epidemiological studies. Since rabies infection can be acquired through organ transplants, molecular assays can also be of use to test donors who are at risk of rabies. Real-time PCR can also be used for quantification of viral RNA to assess the viral load, disease progression and efficacy of experimental therapeutic approaches [63, 73] and may be valuable as newer treatment options for rabies are explored in future.

However, a major limitation of these assays is the need for stringent quality control measures to avoid false positive results and the lack of international standards and universal protocols to be used for diagnosis. Currently, molecular assays are not recommended for routine postmortem diagnosis of rabies; if brain tissue is available FAT should be performed. However, they can be used for antemortem diagnosis of human rabies and for epidemiological surveys in laboratories with strict quality control procedures and with experience and expertise in using such techniques [1].

### 4.3. Demonstration of Antibodies

Though the RFFIT is considered the gold standard to assess the rabies viral neutralizing antibodies, it has several drawbacks as mentioned earlier. Wright et al. investigated antibody neutralization of *Lyssaviruses* using lentiviral pseudotypes. Pseudotypes, which are viruses that carry the genome and core of one virus and the envelope of another, are a safer alternative to live virus neutralization tests and can be used for rabies surveillance studies [74].

 Enzyme-linked immunosorbent assay (ELISA) based methods are being used as an alternative to RFFIT. Though they are only antigen-binding assays and not functional assays which can detect neutralizing antibodies, unlike RFFIT, they are a simple, easy, safer, and rapid alternative to RFFIT. Assays that do not require live virus and high-containment facilities and produce rapid results have been validated and found to correlate well with RFFIT [75, 76]. An ELISA based on monoclonal antibodies to rabies nucleoprotein (N) and glycoprotein (G) was developed to detect rabies specific immune complexes in CSF samples for rapid antemortem diagnosis of human rabies [77]. A second generation ELISA (Platelia Rabies II) developed for detection of glycoprotein antibodies in human serum and CSF samples has undergone a multicentre evaluation. It has been found to correlate well with RFFIT and can be used in laboratories which do not have virus and cell culture facilities [78]. Despite several advantages, ELISA tests have been reported to have lower sensitivity than neutralization tests [79–81]. A new ELISA based method using electrochemiluminescence for detection of rabies glycoprotein antibodies in human and animal sera demonstrated a higher sensitivity than conventional ELISA methods [41]. A rapid neutralizing antibody detection test (RAPINA) developed using the principle of immunochromatography was found to be an easy and rapid method for qualitative and semiquantitative detection of rabies neutralizing antibodies in humans and dogs [82]. A simple and rapid latex agglutination test for detection of rabies specific antibodies has also been described [83].

Recent advances in genetic engineering have led to further improvement in ELISA based assays. A novel double sandwich ELISA utilizing recombinant antigen preparation, for detecting antibodies in dogs and other species, was developed recently [84]. Nimmagadda et al. have described for the first time the use of a recombinant diabody in the development of an ELISA for quantification of rabies viral glycoprotein content in human rabies vaccines incorporating the PV strain of rabies virus and its comparison with the NIH mouse protection test [85]. Assays developed using this technology may have many promising diagnostic applications for rabies in future.

### 4.4. Proteomics and Metabolomics

A recent study reported quantitative proteomic analysis in human brain tissues obtained at autopsy from confirmed cases of encephalitic and paralytic rabies to identify signature proteins that are differentially regulated using high resolution mass spectrometry. Several proteins were differentially expressed, which included karyopherin alpha 4 (KPNA4) overexpressed only in paralytic rabies, calcium calmodulin dependent kinase 2 alpha (CAMK2A) which was upregulated in paralytic rabies, and glutamate ammonia ligase (GLUL) which was overexpressed both in paralytic and encephalitic rabies. These molecules need to be further investigated in body fluids like cerebrospinal fluid in a larger cohort of rabies cases to determine their potential use as antemortem diagnostic biomarkers in rabies [86]. Another recent study on metabolomics of cerebrospinal fluid from humans treated for rabies using proton nuclear magnetic resonance (^1^HNMR) spectroscopy identified several metabolites that differentiated rabies survivors from those who subsequently died [87]. Further studies on rabies metabolomics may provide new insights into diagnostic and prognostic significance of these tests and mechanisms of rabies pathogenesis, which may guide future therapeutic interventions for rabies.

## 5. Conclusions

Rabies still remains one of the most neglected zoonotic diseases worldwide. The low level of commitment to rabies control is partly attributable to lack of accurate and extensive surveillance data to indicate the disease burden, frequent misdiagnosis of rabies, and an absence of intersectoral coordination. Traditional methods for antemortem and postmortem rabies diagnosis are fraught with several limitations. Though rabies is almost always fatal, a few human survivors have been reported in recent years. Hence an early, antemortem laboratory confirmation of rabies provides an impetus for clinicians to attempt experimental therapeutic approaches, especially in patients with paralytic rabies who may have a longer survival. Recent advances in diagnostic methods, including molecular methods, complement the conventional diagnostic approaches and have a potential to revolutionize rabies diagnosis. Though financial and logistical barriers may prevent the routine use of molecular diagnostic assays, the cost/benefit ratio should still be measured. The rapid reduction in turnaround time and cost with these tests indicates that in the near future they will become a viable technology in diagnostic and reference laboratories globally [32, 88].
